# Human organoids: Fit for drug discovery?

**DOI:** 10.1016/j.stemcr.2026.102898

**Published:** 2026-05-07

**Authors:** Annika Wittich, Kim Krieg, Philip Gribbon, Jakob J. Metzger, Alessandro Prigione, Ole Pless

**Affiliations:** 1 Fraunhofer Institute for Translational Medicine and Pharmacology ITMP, Discovery Research ScreeningPort, 22525 Hamburg, Germany; 2 EU-OPENSCREEN, Robert-Rössle-Street 10, 13125 Berlin, Germany; 3 Max Delbrück Center for Molecular Medicine (MDC), Quantitative Stem Cell Biology, 10115 Berlin, Germany; 4 Department of General Pediatrics, Neonatology and Pediatric Cardiology, Medical Faculty and University Hospital Düsseldorf, Heinrich Heine University Düsseldorf, Düsseldorf, Germany

**Keywords:** organoids, human pluripotent stem cells, three-dimensional, drug discovery, assay development, small molecules, high-throughput screening, hit validation

## Abstract

Organoids are self-organizing three-dimensional (3D) *in vitro* tissues derived from pluripotent stem cells (PSCs) that recapitulate key structural and functional features of human organs. Their multicellular architecture and physiological relevance make them promising new approach methodologies (NAMs) for disease modeling, drug discovery, and toxicity testing. However, their reliability and scalability for compound screening remain under evaluation. This review summarizes current human PSC-derived organoid screening strategies, highlighting available readouts, related machine learning methods, and their potential advantages over traditional screening models. We also discuss major challenges, including assay robustness, throughput limitations, and the need for standardized protocols. Advancing validated and scalable approaches will be essential for integrating organoids into pharmaceutical development and improving the translational success of drug candidates.

## Graphical abstract

**Figure:**
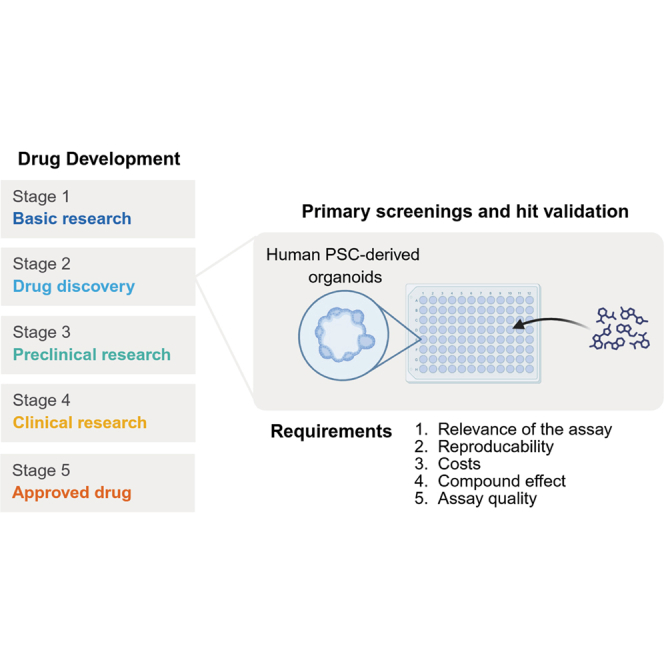

## Teaser

Wittich et al. provide an overview of human PSC-derived organoid screening technologies and outline key considerations for integrating them into drug discovery pipelines. As new approach methodologies (NAMs), organoids have the potential to reduce or replace animal models by enabling the study of cellular responses to pharmacological interventions within a human-relevant physiological context. This review surveys the current landscape of human PSC-derived organoids in compound screening and examines available readouts and analysis techniques, including the application of machine-learning to support modern drug discovery.

## Introduction

The first report of self-organized “organotypic” three-dimensional (3D) cortical tissue derived from mouse and human embryonic stem cells (ESCs) dates to 2008 (Eiraku et al., 2008). The term “organoid” was then introduced in 2009 to describe 3D intestinal structures derived from murine adult intestinal stem cells that can contain all the differentiated cell types of the epithelium and the architecture of intestinal crypts found in mammals (Sato et al., 2009). Organoids are 3D cellular models derived from self-organization processes and can recapitulate the cytoarchitecture and certain functional aspects of human organs (Lancaster et al., 2013; Sato et al., 2009). In contrast to two-dimensional (2D) cultures, this spatial confinement can recreate dynamic cell-cell interactions and mechanical properties of human tissues/organs (Anlaş and Nelson, 2018; Da Silva André and Labouesse, 2024). Whereas tissue stem cell-/adult stem cell-derived organoids can mimic organ regeneration and maintain their native tissue identity, the use of human pluripotent stem cells (PSCs) allows for following developmental trajectories through *in vitro* differentiation, thereby enabling the generation of diverse types of human organoids (Artegiani and Hendriks, 2025; Kim et al., 2020). To date, many differentiation protocols have been published, describing the generation of organoid models representing several human body tissues, including liver (Takebe et al., 2013), brain (Lancaster et al., 2013), kidney (Morizane et al., 2015; Takasato et al., 2015), lung (Dye et al., 2015), colon (Múnera et al., 2017; Spence et al., 2011), and heart (Drakhlis et al., 2021; Ho et al., 2022). Their applications in developmental studies, disease modeling, personalized medicine, and drug development have been extensively highlighted in the literature (Dutta et al., 2017; Vandana et al., 2023; Wang et al., 2025).

**Figure 1:**
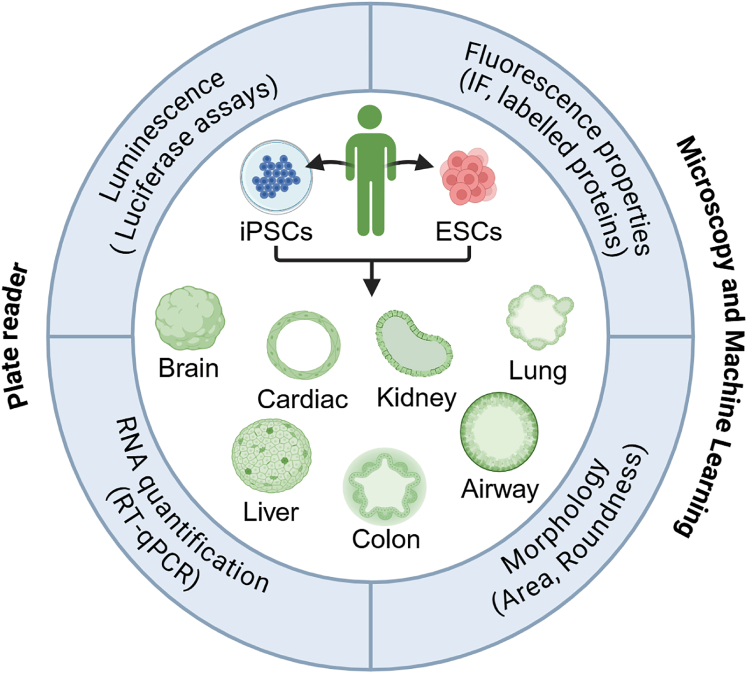
Readout methods in organoid applications for drug discovery Schematic representation of human organoids, derived from either ESCs or iPSCs. ESCs are extracted from a blastocyst, whereas iPSCs are generated from somatic cells. Both ESCs and iPSCs are able to differentiate into the three germ layers (ectoderm, mesoderm, and endoderm) and can give rise to self-organizing 3D organoids. PSC-derived organoids can be used for compound screens, including the readouts and methodologies indicated on the border of the circle.

In this review, we will focus on human organoids derived from PSCs, either from induced PSCs (iPSCs) or ESCs. PSCs provide an unlimited source of derived cells and have the potential to differentiate into cell types of disease relevance (Figure 1). Due to their high proliferation rate, PSCs enable substantial cell yield for large-scale organoid production required for compound testing. By maintaining the genetic background of patients or healthy donors, they also allow addressing individualized compound responses in a defined diseased or healthy context. We here focus on the following organoid types, which have been applied successfully to answer questions related to drug discovery: colon, lung, airway, heart, kidney, liver, and various brain regions such as forebrain, midbrain, cortical brain organoids, and brain neuruloids.

With advancing technology, creating organoids that accurately model specific tissues in terms of cellular composition and function relevant for drug discovery approaches is becoming increasingly feasible. Brain organoids derived from human PSCs, for example, provide a promising platform for modeling neurological disease and testing therapeutic interventions, through the generation of accessible, self-organized neuronal cells that can mimic the (patho)physiology of the human brain (Birey et al., 2017; Lancaster et al., 2013). Given the brain’s complexity, different models have been developed to focus on specific regions, thereby reducing heterogeneity (Cederquist et al., 2019). Forebrain organoids primarily model anterior brain regions such as the cerebral cortex and basal ganglia (Choe et al., 2025), midbrain organoids focus on regions rich in dopaminergic neurons (Fiorenzano et al., 2021), and cortical organoids replicate the layered structure and cellular diversity of the cerebral cortex, responsible for higher cognition (Zhang et al., 2023). Importantly, brain organoids can contain electrophysiologically active neurons (Chung et al., 2022; Paşca et al., 2015). Neuruloids are organoids that simulate the entire ectodermal layer of the human embryo, including the neural, neural crest, placode, and epidermal tissues (Britton et al., 2019; Haremaki et al., 2019), enabling the study of human development and drug responses in otherwise inaccessible environments. Overall, human PSC-derived organoids show significant promise and advantages in physiological relevance compared to 2D models. However, they remain limited in their application to drug discovery compared to native tissues. Organoids often lack vascularization, neural and immune interactions, microbiomes, and full cellular diversity and functionality (Chang et al., 2021; Ouchi and Koike, 2023). Additionally, they lack consistent cellular composition and exhibit considerable variability in formation, morphology, and function, which often hampers their use in drug discovery due to variability in self-organization and cell fate decision (Hofer and Lutolf, 2021).

**Figure 2:**
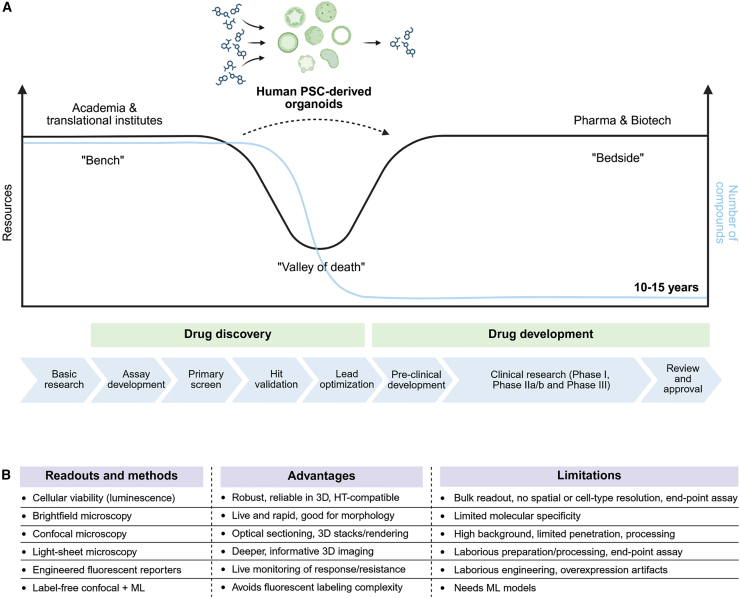
Implementation of PSC-derived organoids in the drug discovery process (A) The drug development process can be divided into different phases: basic research, drug discovery (assay development, primary screen, hit validation, and lead), pre-clinical development, clinical research (Phase I, Phase IIa/b, and Phase III studies), and review and approval. There is a high attrition rate of compounds during this process due to drug failure in early clinical development. Scalable and validated human PSC-derived organoids applied in drug screens can help bridge the drug development “valley of death” by providing meaningful readouts and increasing confidence in predictive value. Broader use can overcome academic resource limits and industry hesitancy, driving improvements in standardization, automation, and translational robustness. (B) Available readouts and methods used in combination with human PSC-derived organoids for drug discovery, summarizing their respective advantages and current limitations. HT, high-throughput; ML, machine-learning.

*De novo* drug development aiming to identify, develop, and market new therapeutics is a long and expensive process that usually takes 10–15 years with costs of up to 2 billion USD (Hinkson et al., 2020). This process can be divided into different stages: (1) basic research, where the underlying disease etiology and progression are studied; (2) drug discovery, which involves identification of disease modulating agents, for example small molecules or antibodies; (3) preclinical development, focusing on elucidating the mode of action of leads and drug candidates and efficacy and toxicity across various *in vitro* and *in vivo* models; (4) clinical research (phase I, phase IIa/b, and phase III studies) in humans; and (5) review and approval by regulatory bodies (e.g., Food and Drug Administration [FDA], European Medicines Agency [EMA], or others) (Singh et al., 2023) (Figure 2A).

Drug discovery is the first step in drug development, involving the process of identifying and validating new potential drugs for a specific intended use (Hughes et al., 2011; Zhou and Zhong, 2017). In the hit finding stages of drug discovery, complementary approaches can be applied, including target-based and phenotypic-based assays. With the advancing field of artificial intelligence (AI), AI-guided drug discovery has very recently emerged as a powerful tool, offering the potential to accelerate key aspects of hit finding and validation (Zhang et al., 2025a). Target-based screening aims to identify drugs that affect single genes or molecular mechanisms (the target) to specifically treat the deficit causing the disease (Samsdodd, 2005). In contrast, phenotypic screening for hits and leads seeks to modulate a disease-specific trait (phenotype) with compounds tested in cells or organisms to determine whether a particular molecule exerts the desired effect, potentially in the absence of information about the underlying target (Horman, 2016; Inak et al., 2017; Khurana et al., 2015). Despite major progress in understanding biological systems through technological advances, a significant challenge in drug discovery remains the high attrition rate (Singh et al., 2023). The failure rate of drug candidates after entering clinical testing is approximately 90% (Sun et al., 2022). One reason for this low success rate is believed to be the limited predictive value of preclinical *in vivo* models (e.g., mice, zebrafish, or fruit flies) and more simplified *in vitro* models (e.g., 2D HeLa and A549 cells) of human disease, which may lead to reduced drug efficacy or unfavorable safety profiles (Harrison, 2016; Paul et al., 2010).

To effectively model human diseases, cellular systems must be designed to reflect the underlying molecular features seen in patients, such as the genetic background, post-translational modifications, and pathway perturbations, as these factors represent the mechanistic drivers of pathology. Applying patient-derived cells helps ensure the model reflects not just superficial phenotypes but also the underlying biology that influences disease onset, progression, and treatment outcomes.

In contrast to PSC-derived 2D cellular models, PSC-derived organoids enable the assessment of multicellular responses and cell-cell interactions within a human genetic background, making them a valuable model for understanding the effectiveness and safety profiles of compounds that cannot be reliably predicted using non-human or 2D preclinical models (Hay et al., 2014; Kola and Landis, 2004; Paul et al., 2010; Seyhan, 2019). Importantly, organoids have the potential to decrease reliance on animal experimentation and support the 3Rs principles (replace, reduce, refine) (Russell and Burch, 1959). In recent years, significant efforts have been made to develop more predictive methods, based on human 2D and 3D *in vitro* models for phenotypic screening (Han, 2023; Zushin et al., 2023). In 2022, the FDA in the United States approved “alternatives to animal testing for purposes of drug and biological product applications,” including cell-based or computational models (https://www.congress.gov/bill/117th-congress/senate-bill/5002). Recently, both the FDA and the EMA outlined a roadmap to reduce animal testing through new approach methodologies (NAMs), where the FDA is initially focusing on monoclonal antibodies to limit animal use in preclinical safety testing (https://www.fda.gov/news-events/press-announcements/fda-announces-plan-phase-out-animal-testing-requirement-monoclonal-antibodies-and-other-drugs) and the EMA decided to foster regulatory acceptance of NAMs (https://www.ema.europa.eu/en/documents/report/new-approach-methodologies-eu-horizon-scanning-report_en.pdf). NAMs include *in silico* (computer modeling), *in chemico* (chemical reactions), and *in vitro* (cell- and tissue-based) methods. Among *in vitro* systems, organoids and organ-on-a-chip (OoC) systems will play a key role in the future (Edwards et al., 2025; Park et al., 2024; Svendsen, 2025). Following extensive efforts to increase robustness and reliability, OoC are starting to be employed in regulatory toxicity tests (https://www.fda.gov/news-events/press-announcements/fda-announces-plan-phase-out-animal-testing-requirement-monoclonal-antibodies-and-other-drugs) (Leung et al., 2022). However, despite their great potential, organoid-based readouts are not yet routinely used in drug development, indicating that many critical challenges must still be addressed. A limitation of many current organoid models is the absence of functional vascular and immune components, which can restrict their ability to capture certain physiological responses to injury and disease. While not all organoid applications require these complex features, incorporating vascularization and immune elements may be important for specific disease contexts where higher levels of tissue complexity are needed (Huang et al., 2021). According to the new FDA guidelines, NAMs do not necessarily need to be fully validated but need to show that they are fit-for-purpose (https://www.fda.gov/media/191589/download). Hence, demonstrating that a specific NAM is appropriate in a defined context of use is a key goal for implementing NAMs into regulatory pipelines. Recent NAM-related breakthroughs include bioprinting and microfluidic technologies, such as OoC to improve vascularization, as well as adding immune cells to simulate tissue-specific immune responses (Chai et al., 2024; Du et al., 2024). Moreover, different organoids can be fused to obtain so-called assembloids, which can be functionally integrated to model interactions between cells of different lineages allowing increased physiological complexity (Onesto et al., 2024). This is the case, for example, of combining brain organoids with microglia or with endothelial organoids and mesenchymal cells to add immune aspects or achieve vascularization-like structures (Song et al., 2019). At the same time, the increased complexity of organoids requires thorough context-specific characterization before they can be applied in drug discovery and screening campaigns. In order to generate reliable physiological models and to interpret any screening outcome, cellular composition, growth behavior, location/cytoarchitecture, cellular trajectories, and levels of maturity need to be studied and must remain reproducible. Although there is significant progress toward developing more physiologically, well characterized organoid systems, their production remains very complex and is not yet fully reproducible and scalable.

In drug finding efforts, a substantial translational gap persists between early-stage discovery in academia and industry-driven development programs. Although many preclinical findings appear promising, a large proportion fail to progress to clinical success. Academic laboratories often lack the financial resources, infrastructure, and automation capabilities necessary for meaningful scale-up and industrial-grade validation. Conversely, industry stakeholders are frequently hesitant to adopt complex and costly model systems that have not yet demonstrated consistent predictive value at scale. The establishment of robust, well-validated organoid-based drug screening applications, along with meaningful readouts (the display of information produced by electronic equipment such as microscopes, sequencing machines, and plate readers), could help bridge this “valley of death,” the gap where many promising preclinical results fail to translate into clinical application (Figure 2A). Broader implementation of organoids in screening campaigns would drive improvements in protocol standardization, automation, and upscaling. Accumulated experience and shared knowledge are likely to further enhance robustness, efficiency, and translational impact.

Here, we provide an overview of case studies covering scaled and reproducible human PSC-derived organoid production, as well as automation systems enabling on 3D model implementation. We describe advanced assay readouts involving disease-relevant endpoints that enable higher-throughput screening, with a specific focus on organoids derived from human PSCs. Additionally, we highlight critical aspects that need to be addressed to enable the reliable integration of human PSC-derived organoids into the overall drug discovery process.

## Organoid specifications for their use in drug discovery

In drug discovery, it is essential to consider both the biological and pharmacological relevance of a screening campaign. The screening model and potential modulators, such as compounds, peptides, or other biological effectors, should be carefully selected based on their physiology, quality, and feasibility. The general requirements for an assay suitable for high-throughput screening include: (1) relevance of the assay, (2) reproducibility, (3) costs, (4) desired (and non-desired) modulator effects, and (5) assay quality (Hughes et al., 2011). For each of these aspects, it is important to understand the potential added value of organoids. In the next section, we will focus on small molecule screening. However, many of the features are equally applicable for studies involving biological-based modulators.

### Relevance of the assay

Several screening campaigns have identified active compounds that may be developed into promising drug candidates using 2D human PSC-based assays, which utilize their ability to differentiate into disease-relevant cell types (Kondo et al., 2017; Lee et al., 2012; Lorenz et al., 2017). Additionally, high-throughput screens were used for efficacy testing in nematodes, fruit flies, and zebrafish to mimic the effects of drugs in the human body and enhance the accuracy of drug screening (Chen et al., 2024). Some of these 2D human PSC-based assay campaigns, especially in the drug repurposing context, have led to active compounds that are progressing to clinical applications, such as retigabine (ezogabine) for amyotrophic lateral sclerosis (ALS), NCT02450552 (McNeish et al., 2015), or sildenafil for Leigh syndrome, NCT06967831 (Zink et al., 2026).

However, when compared to 2D systems, human 3D cellular models have the potential to emulate disease phenotypes more accurately and in a more physiologically relevant manner, as their cellular complexity and cell function are more closely aligned with disease-affected tissue (Kim et al., 2020; Lancaster et al., 2013). For example, qPCR data validated that podocytes derived from iPSC-derived kidney organoids showed enhanced podocyte-specific gene expression compared to 2D cultures, while also preserving polarized protein distribution, thereby demonstrating greater maturation than isolated podocytes or immortalized 2D lines (Hale et al., 2018). Importantly, organoids can maintain the dynamic, reciprocal biochemical, and biophysical interactions between their components, matrix mechanics, and the native niche of parenchymal tissues (Brafman, 2013; Gattazzo et al., 2014; Hofer and Lutolf, 2021), features that 2D PSC models mostly lack. When choosing a cell-based screening model, the assay should provide physiologically meaningful information to address the research question by displaying the disease-relevant phenotype. This means that organoids therefore have the opportunity to replace 2D disease models, when the phenotype cannot be recapitulated in 2D, and when their complexity is needed to answer research questions.

### Reproducibility

A screening assay must be reproducible and replicable, meaning the results should remain consistent regardless of the screening day, assay plates, and stable across different labs, and, when using cells and organoids, independent of their cryopreserved stock or batch of differentiation (Hernández et al., 2022; Kelava and Lancaster, 2016; Phipson et al., 2019). In terms of pharmacology, general best practices for assay development indicate that a coefficient of variation (CV), reflecting the size of a standard deviation relative to its mean, < 20% is acceptable, and CV < 10% is an optimal quality criterion (Sui and Wu, 2007; Zhang et al., 1999; 2012). Moreover, reproducibility metrics such as the IC50 or EC50 shift, the variation in maximal responses, as well as the data of independent repeats, should be assessed and fall within the set CV.

As part of quality control, the intra- and inter-batch variability of parameters like organoid size or adenosine triphosphate (ATP) content should be analyzed and remain stable throughout and between experiments, as demonstrated by Nickels et al. (2020); Renner et al. (2020); Krieg et al. (2025). Some initial approaches have been made to simplify and optimize experimental steps, including reagents and liquid handling steps that substantially reduce variation and facilitate the (semi)-automation of multi-well plate compatible protocols (Boussaad et al., 2021; Brandenberg et al., 2020; Renner et al., 2020). Automated processes can provide superior control of liquid handling processes, such as medium exchanges and addition of detection reagents, ensuring consistency and potentially enhancing the quality of screening assays using organoids. However, while automation can significantly improve throughput and standardization, it also introduces an additional layer of technical and logistical complexity. Automated workflows may give rise to new sources of variability, such as uneven extracellular matrix coating, non-uniform cellular distribution during seeding, or mechanical stress that can dislodge cells and compromise tissue integrity. Moreover, automated systems currently have limited ability to perform context-specific adaptations, as they typically execute protocols as defined sequences. This limitation is particularly relevant for complex assays running over multiple days, where unexpected deviations still require expert oversight. In addition, the implementation and maintenance of automated platforms are associated with substantial financial investment, specialized infrastructure requirements, and the need for technical expertise, all of which can represent significant practical hurdles, particularly for smaller laboratories.

### Costs

When working with organoids, costs for production, maintenance, and data storage should be considered. Indeed, implementing organoid models is relatively expensive compared to 2D approaches, fly, yeast, or worm models (Kim et al., 2020). Additionally, drug screening is standardized for 384- and 1536-well plates, but some organoids grow too large to fit in these well formats and require larger volumes of medium provided by 96-well or 48-well plate formats (Giorgi et al., 2024), which results in higher costs per data point. Due to their complexity and 3D architecture, a large amount of data is generated (e.g., microscopic images and sequencing data), and costs for storage, computing power, and personnel can be high. Although initial expenses may be higher compared to other research models, a potential advantage is that organoids could ultimately lower overall costs by decreasing the need for downstream validation studies in expensive animal models. A typical 2D cell culture data point might cost on the order of several USD, whereas currently a PSC-derived organoid data point costs a multitude of this, reaching up to double or even triple digit USD, mainly due to specialized, expensive reagents, e.g., extracellular matrices (ECMs), growth factors (which are already needed for PSC maintenance), labor-intensive work, long culture times ranging from weeks to months (and thereby increased reagent consumption), and limited automation solutions resulting in lower throughput. These investments have to demonstrate added value in most organizations. Scale and automation represent critical inflection points in the cost curve. With continued automation, standardization, and miniaturization of assay formats, higher-throughput screens (> 1,000 compounds) using organoids could become more feasible (Kim et al., 2026).

### Compound effect

Test compounds need to be soluble in the chosen medium, physically stable, and should not interfere with the assay readout, such as chemically modified proteins. For cell-based assays, compounds must be permeable when the target is located within the cell, non-toxic at screening concentrations, non-mutagenic, and, in order to be developed into an oral treatment, bioavailable (Zhang et al., 2012). Some organoids require hydrogels, which can affect compound diffusion and activity because of their physical barrier effect, increasing the risk of false-negative results in drug testing (Cameron et al., 2023; Mahdieh et al., 2022). Before testing a larger set of compounds, it is necessary to determine the appropriate concentration and incubation time that yields an acceptable hit rate using smaller pilot screens. Notably, the effect size achievable by compounds can differ between 2D and 3D systems. One study found that compounds that promoted iPSC-derived cardiomyocyte proliferation in a 2D format could not be confirmed in a 3D model of human cardiac organoids (Mills et al., 2019). Including a positive control targeting the specific mechanism of action, along with a negative control (often the solvent used for compounds, such as DMSO) on each assay plate enables for the statistical assessment of assay robustness. The plate layout should be evaluated beforehand, as edge effects can occur due to increased evaporation from outer wells during longer assay durations (Gribbon et al., 2005; Iversen et al., 2012; Mpindi et al., 2015).

### Assay quality

Setting up a high-quality assay starts with selecting a reliable screening model. Currently, there are no consensus guidelines or acceptance criteria for organoid-based screening models, which makes it difficult to transition disease models into screening-ready assays. The screening model should undergo thorough validation before initiating the first steps of a screening campaign. This validation includes analyzing cell type composition using methods such as single-cell RNA sequencing (scRNA-seq) to reveal cell type heterogeneity and subpopulations, as well as immunostaining combined with fluorescent microscopy. Additionally, organoids should be evaluated for consistent, tissue-specific functionality. For example, neuronal activity can be measured using multi-electrode array (MEA) recordings or patch-clamp techniques. Cardiac tissue can be tested for contractile force, while liver organoids can be examined for albumin production using methods such as enzyme-linked immunosorbent assays (ELISA) (Tanimizu et al., 2021). Importantly, donor-derived PSCs should be quality-controlled and cultivated according to the “standards for human stem cell use in research” of the International Society for Stem Cell Research (ISSCR) (https://www.isscr.org/basic-research-standards) to ensure high-quality starting material for organoid differentiation (Ludwig et al., 2023). This includes assessing the pluripotency state through quantitative measures of trilineage marker expression for PSC lines, alongside confirming the loss of markers indicating an undifferentiated state (Ludwig et al., 2023). Furthermore, genetic integrity should be verified through genomic analysis to monitor any cellular changes that could influence interpretation of the drug effects. For screening quality control, the Z′-factor is often used as an indicator for screening and as a parameter to assess assay suitability. It captures both the dynamic range and data variation related to signal measurements of both controls (Zhang et al., 1999). A Z′ > 0.5 is generally considered acceptable, although a Z′ of < 0.5 can be acceptable for cellular assays if the number of hits can be validated in follow-up tests (Bar and Zweifach, 2020). However, the implementation and feasibility of the Z′-factor in drug screening assays using complex model systems, such as organoids, is subject to debate in the literature (Bar and Zweifach, 2020; Booij et al., 2022). In particular, the selection of a threshold for Z′ factor should be made in the context of the unmet need for the assay, e.g., < 0.5 (Francies et al., 2018). Decisions should therefore be made on a case-by-case basis. Additionally to the Z′ factor, other assay parameters like signal-to-blank, signal-to-noise, dynamic range, coefficient of variation (CV), and the strictly standardized mean difference (SSMD) can be utilized for assessing the quality and performance of complex biological assays (An and Tolliday, 2010; Krieg et al., 2025; Zhang et al., 2007).

## Current applications and bottlenecks for 3D drug screening

The development of cell-based assays requires a practical approach designed for the specific application. When moving from model systems to assay development, it is crucial to select a few interpretable measurements that reflect disease-relevant endpoints, and assays must be “fit for purpose” (Engle et al., 2018). Although there are an increasing number of protocols for generating PSC-derived organoids, covering nearly all tissue types, significant challenges remain in adapting 2D cellular assays to 3D models, because of their size and thickness, their inaccessibility to inner cells, and their heterogeneity.

Limitations in assay readout technologies continue to restrict the full potential of organoid systems and limit the amount of information that can be obtained (Hofer and Lutolf, 2021) (Figure 2B). For instance, a standard readout used in compound screening is cellular viability, estimated, for example, by the total ATP content of the cell mass, often quantified with luciferase-based assays. Assessing viability upon compound exposure to determine the window between efficacy and toxicity is crucial for drug discovery and the further development of hit compounds, as the cytotoxic concentration can vary depending on cell type and tissue. Cell viability assays, such as the ATP-based CellTiter-Glo (CTG) reagent, are reliable and robust for 3D models (Zanoni et al., 2016) and compatible with high-throughput screening (Han et al., 2021; Renner et al., 2020; Shinozawa et al., 2021). However, the bulk readouts lack spatial information and cannot distinguish cell-specific effects and morphological changes. To maximize the potential of 3D architectures, additional readouts should complement basic measurements like ATP content or size to provide a deeper understanding of biological processes following compound treatment (Lampart et al., 2023).

Imaging coupled with immunolabeling methods and cell compartment-specific dyes has been essential for revealing the cytoarchitecture of organoids and demonstrating that they faithfully reflect aspects of their *in vivo* counterparts (Rios and Clevers, 2018). Capturing reproducible, image-based differences in profiles between diseased and healthy samples forms the basis of a phenotypic assay used in drug screening. This imaging readout can be a single feature extracted from one image channel or a combination of parameters from multiple channels, which differentiates between diseased and healthy states (Chandrasekaran et al., 2021).

Imaging techniques for organoids include brightfield, confocal, and light-sheet microscopy, each with distinct advantages and limitations. Traditionally, thin section preparation combined with classical immunohistochemistry has been widely used to study tissue architecture in 2D and to observe the distribution of single or multiple markers in tissue (Rios and Clevers, 2018). However, full 3D microscopic visualization remains challenging and is not suitable for high-throughput. Live and rapid brightfield and fluorescence imaging in a few planes are preferred for high-throughput and longitudinal studies to monitor the onset of compound effects. Brightfield microscopy can reveal morphological features such as the size and shape of organoids, making it suitable for screening-compatible plate types. Additionally, confocal microscopy offers a non-invasive method for optical sectioning, producing relatively detailed images of organoids through stacked planes that enable rendering. Nonetheless, challenges remain, including high background noise, limited penetration depth, and the complexity of processing tiled images (Fei et al., 2022).

Additionally, automated imaging is complicated by the variable positioning of organoids along the *x*, *y*, and *z* axes. The use of engineered reporter cell lines, which intrinsically express a fluorescent protein, allows for live imaging and monitoring of drug responses, resistance, or relapse (Bury et al., 2024; Sarrafha et al., 2021). However, their application is limited by the labor-intensive process of fluorescent reporter engineering, the restricted number of fluorescence channels, and potential effects of protein overexpression (Jiang et al., 2008). Therefore, label-free confocal imaging methods utilizing machine learning (ML)-based algorithms can bypass the complexity of fluorescent labeling in PSC-derived organoids (Kok et al., 2025; Serafini et al., 2024).

To overcome the challenges of limited tissue penetration and high scattering, light-sheet microscopy offers a more effective and informative solution. However, it is less suitable for high-throughput applications due to the labor-intensive process of sample preparation and image analysis. Since organoids often exceed the microscopic field of view at the necessary magnification, reconstructing single images with 3D rendering software becomes essential. Even with light-sheet microscopy, segmenting and quantifying individual cells for downstream data analysis remains difficult. Tissue clearing is crucial for achieving the cellular resolution needed to apply segmentation and quantification algorithms effectively (Dekkers et al., 2019). Tissue clearing involves techniques that make biological samples transparent, allowing deep imaging of large tissue volumes (Richardson et al., 2021). While several clearing protocols are available, they are generally very time-consuming and often rely on toxic chemicals (Tian et al., 2021).

Furthermore, organoids often depend on animal-derived ECMs. While these support cell growth and differentiation, they are frequently chemically undefined and vary between batches, which hampers reproducibility. This reproducibility can be improved by evaluating multiple ECM batches, selecting the most consistent one, and securing a sufficient supply for long-term experiments. Additionally, physical properties such as temperature sensitivity and high viscosity pose significant challenges for integration into automated liquid handling systems used in scale-up screening applications (Renner et al., 2021).

## Organoids for primary drug screens

Primary screenings are the initial tests conducted in a screening campaign and are essential in the drug discovery process, as they reduce the number of test substances. The goal of the primary assay is to identify hits, which are substances that demonstrate the desired effect and may be biologically active chemical entities (EU-OPENSCREEN_HTS_QC_General_Guidelines200713.pdf) (Hughes et al., 2011).

In the following, we highlight studies using PSC-derived organoids within early-stage drug discovery and employing current screening-compatible readout formats. Some studies have already demonstrated the potential of their developed organoid models for compound testing by administering a single compound (Hale et al., 2018; Lane et al., 2020; Takasato et al., 2015; Winanto et al., 2020). Others confirmed that human PSC-derived organoid models enable a robust, reproducible, and scalable platform supporting drug discovery (Renner et al., 2020). Here, we provide examples of organoids in primary drug screening and hit validation assays, which ultimately overcame the bottlenecks associated with organoids (Table 1).

**Table 1:** Overview of human PSC-derived organoids utilized for primary compound screens and hit confirmation using a multi-well plate format and a screening-compatible readout

Screening type	Tissue	Organoid	Disease/application	Multi-well format	Compounds	Readout	Methodology	References
Primary screen	Airway	ESC-derived airway organoids	SARS-CoV-2	384-well	1,126 compounds at 10 μM; hit: GW6471	IF for SARS-CoV-2 N (infection rate)	Confocal microscopy	Duan et al. (2021)
	Liver	iPSC-derived liver organoids	Liver injury	384-well	238 compounds, 4 concentrations	Fluorescent intensity of bile acid CLF (uptake); luminescence CTG (viability); organoid size	Fluorescence imaging and brightfield microscopy; luminescence	Shinozawa et al. (2021)
	Lung	iPSC-derived lung organoids	SARS-CoV-2	384-well	∼1,300 compounds at 10 μM	Luciferase assay (activity) and CTG (viability)	Luminescence	Han et al. (2021)
	Brain	ESC-derived brain organoids	Huntington	96-well	1,065 compounds at 10 μM	IF for PAX6 (marker of NPCs) and Phalloidin (F-actin) and DAPI staining (Nuclei)	Confocal microscopy; classifier and autoencoder for analysis	Metzger et al. (2022)
	–	iPSC-derived cortical organoids	Teratogenic risk	24-well	298 compounds at 1 µM	Organoid size (area)	Bright-field live imaging	Narazaki et al., 2025
	Kidney	ESC CRSIPR-Cas9 edited kidney organoids	Autosomal dominant polycystic kidney disease (ADPKD)	96-well	247 compounds at 1 μM	Cyst outgrowth area	Brightfield live imaging	Tran et al. (2022)
Hit confirmation	Colon	iPSC-derived colonic organoids	Familial adenomatous polyposis (FAP) inherited colonic cancer	6-well	Geneticin, XAV939, and rapamycin	IF for CDX2 (posterior marker), CCND1, Ki67 (cell proliferation) in CDX2+ cells; quantification of RNA target genes	Confocal microscopy, RT-qPCR	Crespo et al. (2017)
	–	ESC-derived colonic organoids	SARS-CoV-2	24-well	GW6471	Quantification of viral RNA; IF for SARS-CoV-2 N (infection rate)	Confocal microscopy, qPCR	Duan et al. (2021)
	–	iPSC-derived colonic organoids	SARS-CoV-2	24-well	10 μM imatinib, 3 μM mycophenolic acid, 4.5 μM quinacrine dihydrochloride	IF for SARS-CoV-2 N and RT-qPCR analysis for viral N transcripts	Confocal microscopy, qPCR	Han et al. (2021)
	Cardiac	ESC-derived cardiac organoids	Cardiac disease	96-well	105 compounds in 3 concentrations	IF for Ki67 (pro-proliferative) and Hoechst 33342 staining (nuclei); force of contraction	Confocal microscopy, live imaging	Mills et al. (2019)
	Kidney	ESC CRSIPR-Cas9 edited kidney organoids	Autosomal dominant polycystic kidney disease (ADPKD)	96-well	9 hits in DRC (0.1, 1, and 10 μM)	Cyst outgrowth area	Brightfield live imaging	Tran et al. (2022)
	Brain	iPSC-derived cortical organoids	Brain metastasis	96-well	7 compounds at 10 μM	Fluorescent intensity of reporter-engineered melanoma cells; CTG (viability)	Fluorescence imaging and live brightfield microscopy; luminescence	Krieg et al. (2025)
	–	iPSC-derived midbrain organoids	Leigh syndrome	96-well	2 compounds (sertaconazole and talarozole)	IF of neuronal marker (MAP2, TH, SMI312; CTG (viability)	Fluorescence imaging; luminescence; absorbance	Menacho et al., 2026
	–	iPSC-derived forebrain organoids	Zika virus infection	96-well	Hippeastrine hydrobromide (HH), amodiaquine dihydrochloride dihydrate (AQ)	IF for ZIKV E (infection), Ki67 (cell proliferation) and DAPI staining (nuclei)	Widefield fluorescence imaging	Zhou et al. (2017)

ESC, embryonic stem cell; iPSC, induced pluripotent stem cell; IF, immunofluorescence; CTG, CellTiter-Glo; RT-qPCR, reverse transcription quantitative polymerase chain reaction.

These studies adapted human PSC-derived organoids for compound screening in a disease-specific context affecting various tissues like airway (Duan et al., 2021), lung (Han et al., 2021), liver (Shinozawa et al., 2021), brain (Metzger et al., 2022), and kidney (Tran et al., 2022) to multi-well formats (96- or 384-well plates). Organoids were either plated intact (Metzger et al., 2022; Narazaki et al., 2025; Shinozawa et al., 2021; Tran et al., 2022) or dissociated (Duan et al., 2021; Han et al., 2021) into uniform cell suspensions to ensure consistent well-to-well readouts. This enabled screening libraries ranging from ∼250 to > 1,300 compounds. Importantly, organoids retained key human cell types and gene-expression profiles that mirror adult tissues, e.g., ACE2^+^ ciliated airway cells (Duan et al., 2021), AT2-like lung cells (Han et al., 2021), hepatocyte-like cells with bile transport (Shinozawa et al., 2021), early cortical progenitors (Metzger et al., 2022; Narazaki et al., 2025), or nephron-like kidney structures (Tran et al., 2022). This ensured that screening occurred in a context that recapitulates disease-relevant biology. Various readouts were applied assessing viral infection (Han et al., 2021), liver toxicity (Shinozawa et al., 2021), cyst formation (Tran et al., 2022), teratogenic risks (Narazaki et al., 2025) or neurodevelopmental phenotypes (Metzger et al., 2022) using high-content imaging and plate readers. Shinozawa et al., (2021) normalized the luminescence values for organoid size and number, and a deep-learning model was applied to classify phenotypes objectively, enabling unbiased, quantitative hit identification (Metzger et al., 2022). Overall, these studies demonstrate that organoids can function as first-line screening systems by uniting disease-relevant biology, along with scalable, quantitative workflows. Compared with 2D models, they capture tissue architecture, multicellular interactions, and disease phenotypes like viral tropism, bile transport, neurodevelopmental patterning, or cystogenesis, thereby improving the predictive power of primary drug discovery screens. The number of compounds screened to date, however, remains far below the throughput required to evaluate a chemically diverse library for the discovery of novel disease-associated targets or the identification of new chemical entities suitable for intellectual property protection. For organoid platforms to be competitive in primary screening campaigns, they must be scalable to enable screening of several thousand compounds in a robust, reproducible, and cost-effective manner.

## Organoids for hit validation

Following a primary screen, in which compounds are usually applied in singlicates, identified hits need to be confirmed and prioritized in a hit validation screen. Hit validation consists of a suite of assays designed to eliminate false positives and to confirm activity of identified hits in the primary assay, based on their mechanism of action, toxicity, or activity profile (Zhu et al., 2013). As each primary screening assay is unique, so is the follow-up strategy, and a systematic method of hit validation should ideally be in place before the primary screen is completed (Hevener et al., 2018).

On the one hand, organoids were utilized for hit validation following primary 2D drug screens, to confirm the effect in a 3D tissue-specific context (Krieg et al., 2025; Menacho et al., 2026; Mills et al., 2019; Zhou et al., 2017). On the other hand, they have been used for concentration-response testing and secondary assays within the same organoid model, also across diverse genetic backgrounds (e.g., *PKD1* and *PKD2* variants), ensuring that hits were robust and disease-relevant (Tran et al., 2022). Validation relied on whole-mount immunostaining, confocal or high-content imaging, fluorescence quantification, and plate-based assays (e.g., ATP, lactate) (Table 1). Readouts were normalized for organoid size (Menacho et al., 2026) and batch variability (Krieg et al., 2025), enabling reproducible, semi-high-throughput confirmation. Functional readouts included cardiomyocyte proliferation combined with contractile function for cardiac organoids (Mills et al., 2019), melanoma colonization and growth within cortical tissue (Krieg et al., 2025), neuromorphogenesis, and metabolic activity of midbrain organoids (Menacho et al., 2026). These readouts ensured that hits corrected pathological features, not just molecular markers. Some studies showed that compounds effective in 2D failed in organoids or caused unacceptable side effects. For example, compounds that induced proliferation in 2D cardiac cultures failed to do so in immature cardiac organoids, indicating a size effect of compounds in a more complex model (Mills et al., 2019). Moreover, amodiaquine showed anti-Zika virus activity in 2D but was toxic in brain organoids (Zhou et al., 2017). In the context of melanoma brain metastasis, organoids were able to distinguish compounds with anti-metastatic activity and low neurotoxicity (Krieg et al., 2025). Thus, organoids acted as a filter, reducing false positives and identifying the most potent drug candidates. In viral studies, hits from airway or lung screens were validated in colonic organoids, demonstrating efficacy across relevant tissues and strengthening biological conclusions (Duan et al., 2021; Han et al., 2021). This multi-organ approach ultimately increases translational confidence. Overall, human PSC-derived organoids enabled confirmation of therapeutic relevance, detection of tissue-specific toxicity, and assessment of complex phenotypes. Organoids served as physiologically grounded validation platforms that bridge the gap between high-throughput 2D screens and *in vivo* models, potentially reducing the reliance on animal models and increasing the accuracy of drug discovery.

However, examples of drug discovery campaigns using human PSC-derived organoids remain scarce, highlighting the complexity that come along with organoid research.

## Bridging the gap: The promising role of ML

One of the most important aspects of organoid research is the analysis of the images generated by microscopy. Phenotypic screens in a high-throughput, 3D environment require significant technical infrastructure, such as computing power, data storage, and custom analysis pipelines, to extract meaningful insights from the data. Changes in organoid morphology, size, number, architecture, and function provide important information about organoid development and how they respond to changes after drug treatment, but manual image analysis is time-consuming, subjective, and often unable to capture the full 3D complexity of organoids (Bai et al., 2024; Shi et al., 2024).

A promising approach to maximize the potential of organoids in research and drug discovery is to analyze these complex biological models using ML, a subfield of AI that develops algorithms to learn patterns from data. ML-driven image analysis can identify and quantify various organoid features such as size, shape, and structural changes, which are essential for assessing organoid function and maturity. Moreover, ML can reveal image features or phenotypic patterns in screening data that are difficult to detect by manual inspection. By replacing labor-intensive manual scoring with automated algorithms, ML can reduce bias and errors, leading to more reliable and reproducible results in phenotypic assays (Bai et al., 2024). As an example, Lukonin et al. conducted a large-scale phenotypic compound screen in organoids from isolated crypts of mouse small intestine, combining high-content imaging with computational image analysis to quantify multivariate features. These measurements positioned each organoid within a high-dimensional phenotypic landscape, enabling clustering into discrete phenotypic classes that were subsequently consolidated into broader, biologically interpretable categories. By linking these phenotypic classes to specific compound exposures, the study generated a distinct phenotypic fingerprint for every compound, enabling systematic comparisons of phenotypic responses across large chemical libraries (Lukonin et al., 2020). However, this organoid screening was not based on human PSCs. Additional challenges, e.g., size, density, or cellular composition, might limit the transfer of this approach to human PSC-based organoids.

In image-based organoid analysis, ML can be applied at multiple stages of the image-analysis workflow. Features can be extracted using segmentation-based approaches, in which biologically meaningful structures such as nuclei, cells, or organoids are first detected and quantified, or through segmentation-free approaches, where models learn image representations directly from pixel or voxel data. These extracted features or learned embeddings can then be analyzed using ML methods such as supervised learning, for example, to classify phenotypes or predict treatment responses, or unsupervised learning to identify clusters or patterns in the data. While recent deep learning approaches can integrate feature extraction and prediction within a single model, their successful application to complex organoid systems often requires assay-specific training, careful validation, and large image datasets (Metzger et al., 2022). Consequently, although ML holds substantial promise for organoid screening, robust and generalizable analysis pipelines remain an active area of research.

Since organoid imaging faces limitations due to high data dimensionality, acquisition artifacts, low contrast, and bright-field noise, several image segmentation and feature extraction methods have been developed to overcome this specific bottleneck (Louey et al., 2021) (Table 2). General-purpose models like Cellpose (Stringer et al., 2021) and StarDist (Schmidt et al., 2018) are widely used for segmentation of cellular structures such as nuclei or entire cells across diverse imaging conditions. Additionally, there are pixel-classifiers that allow users to annotate pixels within a dataset. Here, ML algorithms learn from these annotations to classify pixels of the remaining dataset. Exemplary open-source tools are Ilastik (Berg et al., 2019) and Trainable Weka Segmentation (Arganda-Carreras et al., 2017). Beyond these, specialized pipelines tailored to organoids have been developed, such as MOrgAna (Gritti et al., 2021), which provides a workflow to segment organoids and quantify their morphological features. Similarly, OrgaExtractor (Park et al., 2023) was developed to segment organoids of various sizes and to track their growth from brightfield images. In contrast, segmentation-free analyses, such as Phindr3D (Mergenthaler et al., 2021) use data-driven voxel-based feature learning to extract high-content features from organoid images. Another platform, Cellos (Mukashyaka et al., 2023), combines classical image processing with ML by using traditional algorithms to identify organoid boundaries and a 3D convolutional neural network (StarDist3D) to segment cell nuclei within organoids. The deep-learning tool deepOrganoid (Powell et al., 2022) predicts cell viability in organoids directly from brightfield images. In the context of drug screening, OrganoIDNet (Ferreira et al., 2025) analyzes time-lapse microscopy of patient-derived tumor organoids to detect treatment responses. These image-based phenotypes often require integrating information across spatial scales, from subcellular changes to whole-organoid structure. ML is particularly useful for such multi-scale integration, as demonstrated for example by the “digitalized organoids” platform, which introduced a multilevel 3D analysis pipeline to concurrently evaluate cellular morphology, cell-cell topology, and whole-organoid shape (Ong et al., 2025).

**Table 2:** Image segmentation and feature extraction methods for phenotypic assessments of organoids

Software tool/model	Type	Application	References
Ilastik	Pixel-classifier	Class-based segmentation by assigning every pixel to a user-defined category	Berg et al. (2019)
Trainable Weka Segmentation	Pixel-classifier	Class-based segmentation by assigning every pixel to a user-defined category as part of the Fiji image processing distribution of ImageJ	Arganda-Carreras et al. (2017)
Cellpose	General-purpose segmentation model	Segments nuclei or whole organoids across diverse imaging conditions	Stringer et al. (2021)
MOrgAna	Pipeline/workflow	Segments organoids and quantifies morphological features	Gritti et al. (2021)
OrgaExtractor	Pipeline	Segments organoids of various sizes and track growth from brightfield images	Park et al. (2023)
Phindr3D	Segmentation-free feature learning	Extracts high-content voxel-based features from organoid images without explicit segmentation	Mergenthaler et al. (2021)
Cellos	Hybrid platform	Classical image processing to find organoid boundaries + 3D CNN (StarDist3D) to segment nuclei	Mukashyaka et al. (2023)
deepOrganoid	Deep-learning tool	Predicts cell viability directly from brightfield organoid images	Powell et al. (2022)
OrganoIDNet	Deep-learning model	Analyzes time-lapse microscopy of tumor organoids to detect treatment responses	Ferreira et al. (2025)
Digitalized organoids	Multi-level analysis platform	Multilevel 3D analysis of cellular morphology, cell-cell topology, and whole-organoid shape	Ong et al. (2025)

Despite these advances, several challenges remain to fully realize the potential of ML in organoid-based screens, with arguably the most important challenge being data variability and scale. The inherent heterogeneity of organoids can confound ML models. Large, diverse, and well-annotated image datasets are needed to train models that generalize across this heterogeneity. For example, OrganoIDNetData contains an open repository of annotated tumor organoid images. Similarly, the MultiOrg project (Bukas et al., 2024) collected a large database of organoid images, allowing for the quantification of annotation uncertainty by including multiple independent labels for organoid images.

Another notable gap is the interpretability of ML models. Complex deep learning algorithms often act as “black boxes,” for which it is difficult to determine which image features drive a particular prediction. However, even when specific features can be identified and their contribution quantified, they do not necessarily translate into meaningful biological interpretation. Segmentation-free architectures face constraints in feature interpretability, because they are learned directly from raw pixels rather than from biologically grounded morphological structures. This makes it difficult to map model-derived features back to specific cellular or subcellular phenotypes (Mergenthaler et al., 2021). This lack of transparency can hinder biological insights and trust in AI-driven decisions. While ML and AI have been used in the context of drug discovery (Catacutan et al., 2024; Jiménez-Luna et al., 2020), this is a promising research direction that remains an emerging approach in organoid screening (Lukonin et al., 2020; Metzger et al., 2022).

## Conclusion and perspectives

Despite several limitations of human PSC-derived organoids for drug discovery, including reproducibility, variability, upscaling challenges, batch-to-batch variations, and data analysis, 3D models present significant potential in drug discovery. The research field of organoids has extended the recapitulation of human tissue to study organ development, model diseases, and examine compound responses in a more physiological environment compared to 2D cellular models, aiming to accelerate drug discovery by making it more relevant and predictive. Developing standardized protocols, automating liquid handling systems, implementing ML strategies, and leveraging new technologies are now essential to overcoming the current limitations.

It is important to highlight that 3D models might not be suitable for all research purposes. Researchers need to carefully evaluate if a 3D model is the best option for their drug discovery study, asking whether it provides more robust evidence and physiologically relevant information compared to 2D models, and if the available analysis tools can extract meaningful data while meeting the quality requirements for primary high-throughput drug screening applications. Additionally, traditional gold-standard methods used in 2D cultures may not directly apply to 3D models, and current readouts often fail to fully capture the complexity of 3D architecture. Although we highlight exemplary studies that used human PSC-derived organoids for primary screens, the number of compounds and readout complexity used in these studies was still relatively low. Upscaled organoids may lack maturation and full functionality, while complex physiological organoids are time-consuming to produce and not yet suitable for screening thousands of compounds. A practical approach in modern drug discovery could be to use cellular models with enough complexity to match the assays to be performed. If 2D models provide sufficient physiologically relevant information, they can be used for large-scale screenings of compounds, with organoids then employed to validate a smaller list of hit compounds, potentially reducing the failure rate in drug development.

In the past years, OoC technology, defined as microfluidic devices, containing living engineered organ substructures in a controlled microenvironment (Moruzzi et al., 2023), has supported drug screening, especially for toxicity assessment (Akarapipad et al., 2021; Schneider et al., 2021). Although a considerable variety of OoCs have been developed, the heart-, liver-, kidney-, and brain-on-a-chip are the most commonly investigated organs in the domain of drug screening, as they are the four major target organs of drug toxicity (Wang et al., 2023a). OoC mimic the microphysiological environment cells experience in a tissue, including the vasculature-like perfusion (Cipriano et al., 2022) but lacking the 3D structural architecture and cell-cell interactions. While OoC aims to replace animal toxicity studies, organoids may have a specific role in functional validations. The value of using organoids as physiologically relevant and complex NAMs in validation steps could possibly replace the use of extensive animal experimentations. However, organoids are still far from having the complexity, reproducibility, and functionality needed to completely replace animal studies before entering clinical phases. Currently, it is still crucial to assess whether findings from an organoid can be recapitulated in the more complex *in vivo* tissue (Taelman et al., 2022). At the same time, animal models may not be available for all possible disorders and could exhibit limitations in recapitulating specific human disease features. Hence, organoids may represent a complementary approach in drug development pipelines that may become increasingly used if it proves to demonstrate practical advantages.

Improving readout technologies is essential not only for drug discovery but also for organoid research overall. Despite progress in recent years, a gap remains between the complexity of organoid models and the analytical tools available. ML could potentially bridge this gap by providing individual downstream analysis tools to maximize the output and insights of phenotypic readouts, enabling strict classification of phenotypes and overcoming challenges associated with multicellular organoids and limited imaging techniques. Ongoing advancements in tissue clearing and high-resolution imaging (Huang et al., 2021) may enable multiparametric phenotypic screening like cell painting—a technique involving the simultaneous staining of various cellular features, capturing thousands of morphological features, including shape, texture, size, intensity, and spatial relationships among organelles to predict the mechanism of action of a perturbing substance (Bray et al., 2016; Pahl and Sievers, 2019; Seal et al., 2025) in the future. “Organoid painting” in combination with deep learning AI algorithms could potentially be a highly informative readout format. Moreover, AI models have the ability to preselect the compounds by predicting targets, pathways, or structures prior to *in vitro* screening. Using cutting-edge ML techniques like transformers, graph neural networks, and diffusion models, AI can forecast targets for future precision therapies and generate candidate compounds tailored to specific patient groups.

Diffusion models show promise in drug discovery to design novel molecular structures *de novo*, complementing traditional medicinal chemistry (Zhang et al., 2025b). Graph neural networks, which model molecules as atom-bond graphs, have improved molecular property prediction and binding affinity estimation (Wang et al., 2023b). Virtual screening, including methods based on AI-informed molecular dynamic simulations (Wang et al., 2024) is about to revolutionize drug screening by enabling the efficient identification of potential hits from extensive chemical repositories while still screening the minimum number of compounds (Gryniukova et al., 2023). AI informed *in vitro* screening workflows configured within tightly linked design/make/test/analyze (DMTA) cycles also enable testing fewer, curated sets of compounds directly in organoid models, reducing the overall number of compounds needed and thereby lowering costs and experimental workload, while maintaining screening success (Zhavoronkov et al., 2026). In a study by Menacho et al., the authors applied a deep-learning approach trained on a human database that did not include cancer cells to predict which gene-expression changes in progenitors would promote effective neural commitment in cells carrying pathogenic variants causative of Leigh syndrome, a currently incurable mitochondrial neurological disease. These gene expression changes were then used to predict repurposable compounds capable of inducing similar transcriptional signatures. This approach allowed the model to identify innovative treatment candidates that were subsequently validated in a focused hit confirmation screen using patient-derived midbrain organoids (Menacho et al., 2026).

To achieve successful employment of brain organoids as NAMs in drug development, appropriate guidelines on model validation, cell cultivation, image acquisition, and data analysis are necessary (Carragher et al., 2018). A thorough review of the legal framework surrounding organoids is still lacking, especially regarding their creation, legal status, and usage. Establishing an organoid data repository would be vital for comparing and assessing organoid quality and donor variability. Sharing data, protocols, and readout methods is essential to promote broader accessibility and collaboration within the research community, while also adhering to the FAIR principles (findability, accessibility, interoperability, and reusability) to improve the reuse of existing data (Wilkinson et al., 2016). To date, a comprehensive database (http://www.inbirg.com/organoid_db/) has been developed for multi-dimensional exploration of bulk and single-cell transcriptome profiles of organoids (Ma et al., 2023). Additionally, an Organoid Cell Atlas is under development within the Human Cell Atlas (Regev et al., 2017), focusing on the single-cell characterization of organoids and complex *in vitro* systems (Bock et al., 2021; He et al., 2024).

In summary, finding the “sweet spot” when choosing a disease model with sufficient complexity while maintaining feasibility for compound screenings is crucial. The demonstration that organoids are fit-for-purpose within a defined context of use is essential to allow their implementation in regulatory settings. At the same time, the case studies presented here highlight the power of human PSC-derived organoid technology and its practical application in drug discovery. The limited number of examples, present to our knowledge, underscores the inherent difficulties associated with PSC-derived organoid systems. The scientific and technological progress in all the mentioned domains will support the transition of novel preclinical organoid models to more standardized applications for drug discovery.

## Acknowledgments

We acknowledge support from the 10.13039/501100001659Deutsche Forschungsgemeinschaft (DFG) (PR1527/6-1, PR1527/15-1, PR1527/14-1, PR1527/13-1 to A.P. and SFB TRR 305, 429280966 to O.P.), the European Joint Program on Rare Diseases (EJP RD) and Federal Ministry of Research, Technology and Space (BMFTR) (01GM2002A to A.P., 01GM2002B to O.P.), the 10.13039/501100000780European Commission’s Horizon Europe Program (101080249 to A.P.), and 10.13039/501100004923AFM-Téléthon (28545 and 29044 to A.P.). Figures were created with BioRender.com.

## Author contributions

Conceptualization, A.W., A.P., and O.P.; writing – original draft, A.W.; writing – review and editing, K.K., P.G., J.J.M., A.P., and O.P.; visualization, A.W.; funding acquisition, A.P. and O.P.

## Declaration of interests

The authors declare no competing financial or commercial interests. A.P. filed patent applications for the use of sildenafil, talarozole, and sertaconazole in mitochondrial diseases.
